# Supplementary material for: Inhibitory Effect of Adsorption of Streptococcus mutans onto Scallop-Derived Hydroxyapatite
Source: Int J Mol Sci. 2023 Jul 12;24(14):11371. doi: 10.3390/ijms241411371 (PMC10379008; doi:10.3390/ijms241411371)
Supplement: Supplementary file 1 [file ijms-24-11371-s001.zip › ijms-2451321-supplementary.pdf]

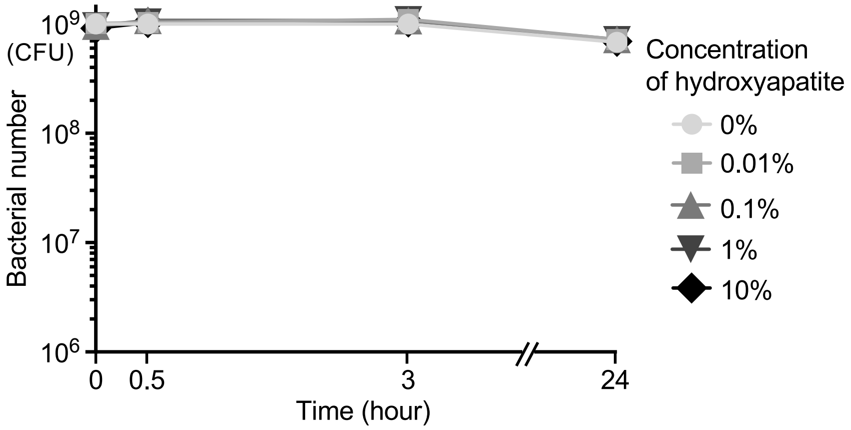

Figure S1. The number of *S. mutans* after the reaction of the bacteria with scallop-derived hydroxyapatite.
